# Libraries

**Published:** 1875-01

**Authors:** 


### LIBRARIES.

We are prepared to supply public and private libraries with all books published in Europe or America. Our prices are precisely those of the publishers, except for large orders; for these we make a fair discount. Of course we do not furnish rare books or such as are out of print, at the publication rates, but our facilities in New York and London enable us to fill orders in the book line, at the lowest practicable prices.

Foreign Periodicals.—We have an office in London from which we send out to American and Canadian subscribers all the European magazines and newspapers at the publishers' rates, with the inland and ocean postage prepaid. Subscriptions for English, French and German magazines, and other periodicals, are received at our New York office. We charge subscribers no profit, as the various publishers allow us a small margin, which compensates us for our trouble.

Our Clubbing Facilities.—We are doing a large business in subscriptions for other publications, which we send out in connection with Hall's Journal, at very low figures. Publishers of other journals recognize us as a live concern, and give us terms which enable us to supply the leading monthlies and weeklies with our magazine for a sum not much greater than is demanded for the publication with which we club. Thus we send either of Harper's periodicals—the *Monthly*, *Weekly*, or *Bazar*—and Hall's Journal, both postage paid, for $5 per year. We supply the *Christian Union*, Mr. Beecher's great weekly, with Hall's Journal, for $4 per year, both postage paid. Read the advertisement of the *Christian Union* in this number.

Address:

THE AMERICAN & FOREIGN PUBLICATION CO.,

137 Eighth street, New York,

Or 17 Henrietta street, Covent Garden, London.

---

All receipts for monies remitted must bear the name of E. H. Gibbs, Treasurer, to be valid.

